# SGLT2 Inhibitors and the Risk of Urogenital Infections: A Concise Review

**DOI:** 10.3390/jcm14061960

**Published:** 2025-03-14

**Authors:** Luminita-Georgeta Confederat, Oana-Maria Dragostin, Mihaela-Iustina Condurache

**Affiliations:** 1 Department of Biomedical Sciences, “Grigore T. Popa” University of Medicine and Pharmacy of Iasi, 700115 Iasi, Romania; mihaela-iustina.condurache@umfiasi.ro; 2 Research Centre in the Medical-Pharmaceutical Field, Faculty of Medicine and Pharmacy, “Dunarea de Jos” University of Galati, 800008 Galati, Romania; oana.dragostin@ugal.ro

**Keywords:** SGLT2 inhibitors, urogenital infections, prevalence, risk factors, clinical impact

## Abstract

Diabetes mellitus has become a major public health problem due to aspects such as an alarming increase in prevalence, the morbidity and mortality associated with its complications and, not least, the economic burden. SGLT2 inhibitors are a relatively new but valuable class of drugs that demonstrated multifaceted effects in addition to hypoglycemic action. Moreover, these drugs demonstrated cardiovascular and renal benefits, even in individuals without diabetes, being recommended by current guidelines to patients with a history of cardiovascular disease, or at high risk for it, as well as to patients with chronic kidney disease. The prescription of this class of drugs is limited by the risk of urogenital infections, despite their multiple demonstrated benefits. Data regarding the prevalence of SGLT2 inhibitors associated with urogenital infections depend on several factors related to the study carried out and to other additional conditions that could precipitate such infections. While SGLT2 inhibitors have a well-established association with the risk of genital infections, the association with urinary tract infections remains controversial and uncertain. This review will be focused on urogenital infections associated with the administration of SGLT2 inhibitors, highlighting their prevalence, risk factors, mechanisms involved, clinical relevance and particularities of management.

## 1. Introduction

Diabetes mellitus is a complex metabolic condition characterized by chronic hyperglycemia which, if poorly controlled, can lead to serious microvascular and macrovascular complications associated with reduced life expectancy [1]. Due to the increasing prevalence of morbidity and mortality attributed to diabetes complications and the economic burden, this disease has become a major public health problem [2]. According to International Diabetes Federation (IDF) statistics, during the last two decades, the prevalence of diabetes has dramatically increased in every region of the world; in 2021, globally, there were 537 million people with diabetes (10.5% of the population); if this trend continues, the predictions show that by 2030, 643 million people will be affected by this disease, and the number of cases will reach 783 million people by 2045 (12.2% of the population) [3]. Related to acute and chronic complications of diabetes, the main cause of morbidity and mortality in patients affected by this condition is represented by different forms of cardiovascular disease, which is 2–4 times more frequent in people with diabetes compared to the general population [4].

In accordance with this alarming increase in prevalence and its devastating complications, several studies have been focused on the development of different classes of drugs in order to achieve and maintain optimal glycemic control. Older antidiabetic agents including metformin, sulfonylureas, thiazolidinediones, meglitinides and dipeptidyl peptidase (DPP) inhibitors were associated with a reduction of microvascular complications, but with no significant effect on macrovascular complications [5,6]. The last two classes of antidiabetic agents introduced in therapy, glucagon peptide-1 receptor agonists (GLP1-RA) and sodium-glucose co-transporter 2 (SGLT2) inhibitors, have proven cardio-renal benefits, which changed the latest guideline recommendations of the American Diabetes Association (ADA), with these drugs being the first-line medication in patients with established cardiovascular disease, heart failure and chronic kidney disease [6,7].

SGLT2 inhibitors are antidiabetic drugs that demonstrate multifaceted effects in addition to hypoglycemic action, such as weight loss, control of blood pressure, improvement of lipid profile and fatty liver disease, as well as the modulation of arterial stiffness and endothelial function [8,9]. Their mechanism of action consists of the inhibition of the absorption of glucose from the proximal kidney tubule, resulting in glycosuria. In normal conditions, the glucose filtered is entirely reabsorbed in the kidney tubule, so the glucose is not present in the final urine. When plasma glucose values are over 180 mg/dL, a value considered the threshold for glycosuria, the filtered glucose is excreted in the urine. On the other hand, studies have suggested that diabetic people present increased expression of SGLT2 in tubular cells, leading to elevated levels of renal threshold of glycosuria and increased glucose reabsorption capacity in the kidney, as an adaptation mechanism of the body to limit energy loss [10,11]. At the moment, there are four approved oral agents from this therapeutic class (Canagliflozin, Dapagliflozin, Empagliflozin and Ertugliflozin), with different levels of selectivity and potency against SGLT [12].

SGLT2 inhibitors proved effective results on glycemic control, assessed through glycated hemoglobin values (HbA1c), fasting plasma glucose and postprandial glucose levels in different trials that studied the effects on glycemic control in treatment-naive patients or when added to metformin, sulfonylureas, thiazolidinediones, DPP inhibitors, GLP1-RA or basal insulin [9]. For example, dapagliflozin proved to reduce HbAc1 levels by 0.82–0.89% after 24 weeks of administration in treatment-naive patients and by 0.84% in patients treated with metformin, the level of HbA1c reduction being associated with the baseline values and greater reductions being observed in patients with higher HbA1c at baseline [13,14]. In addition, dapagliflozin demonstrated a reduction of HbA1c values by 0.82% when added to glimepiride, by 0.97% when added to pioglitazone and by 0.57% as add-on therapy to basal insulin; an important aspect is the fact that the co-administration of dapagliflozin with pioglitazone resulted in less edema and weight gain, while the co-administration with basal insulin led to the reduction of the daily dose by about 2 units and to the decrease in body weight by about 1.6 kg [15,16,17] Concerning the other SGLT2 inhibitors, the studies carried out demonstrated similar results on glycemic control [9].

Considering the fact that cardiovascular disease and chronic kidney disease are the most frequent comorbidities in patients with diabetes, as well as the fact that cardiovascular disease represents the leading cause of mortality in this group of patients, there were several trials with cardiovascular and renal outcomes carried out. The cardiovascular safety profile of empagliflozin was studied through an EMPA-REG OUTCOME trial, which included about 7000 patients with type 2 diabetes cardiovascular disease—coronary, cerebrovascular or peripheral arterial disease randomized to receive empagliflozin or placebo; the results showed that empagliflozin reduced death from cardiovascular causes by 38%, death from any cause by 32% and hospitalization for heart failure by 35%, while myocardial infarction and stroke were not influenced [18]. Similarly, the effects of dapagliflozin on cardiovascular events were studied in the DECLARE-TIMI 58 trial, which included about 17,000 type 2 diabetic patients with established atherosclerotic cardiovascular disease or multiple risk factors for this randomized study to receive dapagliflozin or placebo; the results showed that dapagliflozin reduced cardiovascular death and hospitalization for heart failure by 17% [19]. Finally, canagliflozin reduced the composite 3P-MACE (death from cardiovascular causes, non-fatal myocardial infarction, non-fatal stroke) by 14% in the CANVAS study [20].

The effects of SGLT2 inhibitors on the kidney were studied through three large clinical trials with renal outcomes focused on patients with chronic kidney disease. The CREDENCE trial assessed the renal effects of canagliflozin in adults with type 2 diabetes, macroalbuminuria and eGFR between 30–90 mL/min/1.73 m^2^; the results showed a reduction by 32% of the risk of developing end-stage kidney disease in the canagliflozin group versus the placebo. The effects of dapagliflozin on renal outcomes were studied through a DAPA-CKD trial, which included about 4300 patients with chronic kidney disease, 2/3 with diabetes and 1/3 without diabetes; the results demonstrated a relative risk reduction of 39% for sustained eGFR decline over 50%, end-stage kidney disease and renal or cardiovascular death. Similarly, empagliflozin showed a relative risk reduction of 28% in kidney disease progression with the EMPAREG OUTCOME trial [7].

**Figure 1:**
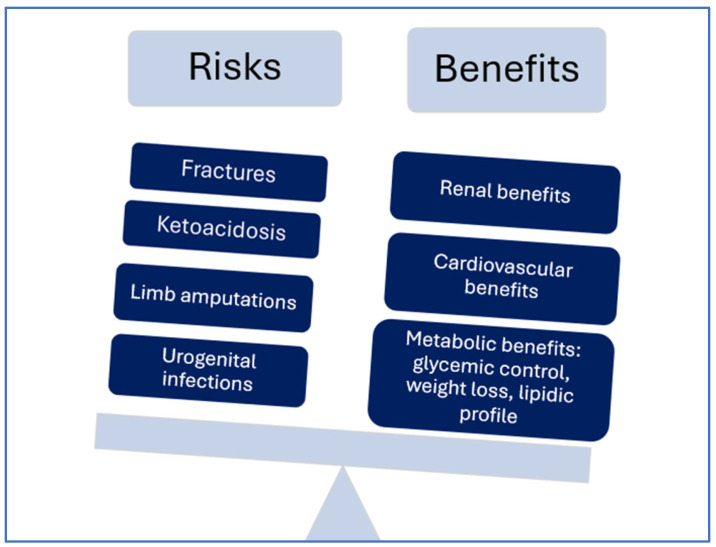
SGLT2 inhibitors between risks and benefits.

Despite the multiple metabolic, cardiovascular and renal benefits of SGLT2 inhibitors, the possible side effects associated with this class of drugs are not lacking, some of them being a result of their mechanism of action and others being reported cases (Figure 1). However, the data concerning the clinical relevance and the prevalence of these side effects are contradictory. Some case reports concluded that treatment with SGLT2 inhibitors may be associated with an increased risk of euglycemic diabetic ketoacidosis, triggered by some factors such as severe insulin deficiency, low carbohydrate diet, alcohol intake, dehydration or some severe diseases [21]. Another important side effect is represented by urinary tract and genital infections, explained through the glycosuria induced by these drugs and the elevated urine glucose levels predisposing patients to this condition; however, the results of the studies showed contradictory data related to this subject [22]. It has been suggested that SGLT2 inhibitors may affect bone density and increase the risk for fracture by altering mineral metabolism, and this risk is higher with canagliflozin versus placebo, while there was no difference with dapagliflozin and empagliflozin versus placebo [23]. Additionally, there was an almost double incidence of lower limb amputations reported associated with canagliflozin compared with the placebo, while no difference was observed for other SGLT2 inhibitors. This side effect could be explained by volume depletion that may affect the perfusion of the limb already with vascular dysfunction [24]. Another concern is related to the increased risk of developing breast cancer in women and bladder cancer in men, one possible explanation being the promotion of tumor growth in the presence of glycosuria and urinary tract infections, but the results of large trials did not confirm this hypothesis [25,26]. Finally, the risk of hypoglycemia associated with this class of drugs seems to be low; the risk is increased when SGLT2 inhibitors are administered concurrently with sulfonylureas or insulin [27]. Concerning the incidence of the mentioned side effects, a meta-analysis of large randomized trials with SGLT2 inhibitors showed that these drugs significantly increased the risk of diabetic ketoacidosis (risk ratio-RR 2.57), genital infections (RR 3.75) and volume depletion (RR 1.14); also, there were increased trends observed in the risk of fractures (RR 1.07), lower limb amputations (RR 1.21) and urinary tract infections (RR 1.07) [28]. A meta-analysis of 40 cohort studies highlighted that SGLT2 inhibitors administration was associated with an increased risk of diabetic ketoacidosis (hazard ratio HR 1.21) and genital tract infections (HR 2.72), while these drugs were not associated with an increased risk of lower limb amputation (HR 1.06) or bone fractures (HR 0.99). Additionally, the risk of hypoglycemia was reduced [29].

From the side effects of SGLT2 inhibitors discussed above, urinary tract infections seem to be the most concerning problem for clinicians, which limits the administration of this class of drugs despite their multiple demonstrated benefits. This review will be focused on urogenital infections associated with the administration of SGLT2 inhibitors, highlighting their prevalence, risk factors, mechanisms involved, clinical relevance and particularities of management.

## 2. Urogenital Infections Associated with SGLT2 Inhibitors

### 2.1. Prevalence

The data concerning the prevalence of genital and urinary tract infections in patients treated with SGLT2 inhibitors vary depending on several factors such as the type of the study carried out, the number of patients included, the inclusion/exclusion criteria, the follow-up period and, not least, additional factors that could precipitate such infections.

A retrospective study carried out in a tertiary care hospital in India that included patients with type 2 diabetes receiving SGLT2 inhibitors for at least 12 months (120 participants) showed that 16.6% of patients presented one or more episodes of genital mycotic infections, while 3.3% patients developed urinary tract infections. The results demonstrated no gender difference related to the occurrence of genital mycotic infections, while urinary tract infections were more prevalent in women. Also, the majority of genital mycotic infections occurred during the first year of treatment and 6.6% of patients presented recurrent mycotic infections. The study did not find any significant correlations between the incidence of genital mycotic infections and factors such as the age of the patients, duration of the disease, other drugs associated with SGLT2 inhibitors, glycemic control, dose and drug from this class [30].

Another prospective longitudinal study developed in a tertiary care center in India that included 80 patients with type 2 diabetes who started the treatment with SGLT2 inhibitors aimed to evaluate the prevalence of genital and urinary tract infections at baseline and after 12 weeks from the initiation. The results showed that asymptomatic bacteriuria was present in 1.3% of patients, while symptomatic urinary tract infections were found in another 1.3% of patients. Related to genital mycotic infections, these were reported in 3.3% of women and 6.7% of men receiving SGLT2 inhibitors. Thus, this study concluded that this class of drug was well tolerated, had a favorable safety profile and the risk of urinary tract infections was minimal [31].

A population-based cohort study carried out over 2 years aimed to assess the risk of severe urinary tract infections in type 2 diabetic patients initiating SGLT2 inhibitors compared to those initiating DPP inhibitors and GLP1-RA; the results showed that the risk of severe urinary tract infections among patients initiating SGLT2 inhibitors was similar to that among patients initiating DPP inhibitors (HR 0.98) and GLP1-RA (HR 0.72) [32]. Similar data were reported by a population-based cohort study carried out in Canada which aimed to assess the risk of urinary tract infections and recurrences associated with the initiation of SGLT2 inhibitors in comparison with DPP inhibitors, GLP1-RA, sulfonylureas, thiazolidinediones and insulin. The study included about 7400 participants and the results demonstrated that the use of SGLT2 inhibitors did not increase the risk of urinary tract infections or their recurrence compared to other non-insulin medications; moreover, the risk of urinary tract infections was lower compared to insulin [33].

A recently published retrospective cohort study carried out in Japan aimed to evaluate the association between SGLT2 inhibitors and the incidence of genital bacterial infections, urinary tract infections and soft tissue infections. The study included about 35,000 patients who were initiated on SGLT2 inhibitors or DPP inhibitors between 2014 and 2020; the results showed that the administration of SGLT2 inhibitors was associated with a decreased risk of urinary tract infections and an increased risk of genital bacterial infections compared with DPP inhibitors, while there was no significant association with soft tissue infections [34].

A systematic review and meta-analysis of 77 randomized controlled trials involving about 51,000 participants evaluated the effects of SGLT2 inhibitors on the development of urinary tract infections and genital infections in patients with type 2 diabetes; the results highlighted an increased risk of genital infections associated with SGLT2 inhibitors versus the control, but no significant difference related to the risk of urinary tract infections [35].

In contrast, a large retrospective cohort study evaluated the risk of genital and urinary tract infections associated with SGLT2 inhibitors compared to DPP inhibitors, sulfonylureas and thiazolidinediones prescribed in addition to metformin in type 2 diabetes patients. The study involved about 107,000 patients who were followed between 2014 and 2017; the results highlighted that SGLT2 inhibitors were associated with an increased risk of genital infections compared to DPP inhibitors (HR 2.39), sulfonylureas (HR 3.23) and thiazolidinediones (HR 3.23). Concerning urinary tract infections, even if HR were lower, the use of SGLT2 inhibitors was associated with a significantly increased risk compared to DPP inhibitors (HR 1.57), sulfonylureas (HR 1.66) and thiazolidinediones (HR 1.69). The results did not show significant differences between drugs from the SGLT2 inhibitors class, both concerning urinary tract infections and genital infections [36]. These findings were in accordance with the results of other large studies; similarly, a retrospective longitudinal cohort study carried out in Australia, a systematic review in China and a retrospective cohort study in the United States have reported that the administration of SGLT2 inhibitors is associated with an increased risk of genital infections compared with DPP inhibitors [32,37].

Not least, although SGLT2 inhibitors have been approved for the treatment of type 2 diabetes, now this class of drug is recommended for the management of other conditions such as heart failure and chronic kidney disease [38,39]. The administration of these agents was hypothesized to increase the risk of urogenital infection, but the incidence rate of this side effect was less studied in patients without diabetes. In this regard, a systematic review and a meta-analysis of 12 randomized controlled trials were conducted that evaluated the urogenital side effects reported in patients without diabetes treated with SGLT2 inhibitors. The results showed that the administration of this class of drugs in diabetic patients was associated with a significantly higher risk of genital infections, but not urinary tract infections, compared to non-diabetic patients; in addition, the risk of genital infections was also increased in individuals without diabetes, but to a lesser extent [40].

### 2.2. Risk Factors

According to the new guidelines, SGLT2 inhibitors are drugs with demonstrated cardiovascular and renal benefits irrespective of the presence of diabetes mellitus, which are recommended for patients with both reduced and preserved ejection fraction heart failure, as well as for patients with chronic kidney disease with eGFR ≥ 20 mL/min/1.72 m^2^ and proteinuria [41,42].

Diabetes, especially when poorly controlled and associated with microvascular complications, is considered one of the most important risk factors for the development of urinary tract infections in patients treated with SGLT2 inhibitors. Related to this, in randomized controlled trials (RCTs) of SGLT2 inhibitors with cardiovascular outcomes, the incidence of urinary tract infections was higher in patients with diabetes; however, a meta-analysis of four RCTs found that there was no difference in the odds of urinary tract infections between diabetic patients and those without diabetes [40,43]. The additional risk factors for urinary tract infections associated with SGLT2 inhibitors include duration of diabetes, age over 65 years, proteinuria, eGFR ≤ 60 mL/min/1.72 m^2^, urinary tract obstruction and the presence of a lower urinary tract infection such as cystitis or bacteriuria at the baseline when initiating this class of drugs [44,45,46]. Concerning microvascular complications of diabetes, diabetic autonomic neuropathy that could lead to an atonic or neurogenic bladder is of particular importance, causing severe upper urinary tract infections [46]. The association between low eGFR and SGLT2-induced urinary tract infections is complex; on the one hand, with worsening renal function, SGLT2-induced glycosuria decreases, so lower eGFR could be protective for infections at this level; on the other hand, urinary tract infections in this group of patients could result from impaired host defense mechanisms with chronic kidney disease, considerations that could apply to other risk factors such as age and proteinuria [42,47]. Not least, SGLT2 inhibitors are frequently co-administered with other classes of drugs in the complex treatment regimens of diabetic patients with cardiovascular and renal disease, and the drug interactions could increase the risk of urinary tract infections. Related to this, drug–drug interaction testing showed that the association of SGLT2 inhibitors with drugs such as DDP inhibitors, statins, angiotensin II receptor blockers and calcium channel blockers increased the risk of urinary tract infections in diabetic patients [48].

Concerning genital mycotic infections, the presence of diabetes is also a risk factor in patients treated with SGLT2 inhibitors, the risk being higher with a longer duration of the disease and poor glycemic control. Additionally, the administration of sulfonylureas or insulin together with SGLT2 inhibitors was associated with a 3-fold higher risk of genital mycotic infection in male patients [43,49]. Other risk factors for the development of this type of infection seem to be gender-dependent: in women, a previous history of genital mycotic infection and obesity could increase the risk and this might be higher in postmenopausal women as a result of diminished immunity in the reproductive tract; in men, circumcision was associated with a lower risk of genital mycotic infections, probably due to improved local hygiene [50,51]. In addition to the mentioned conditions, several factors influence the risk of urogenital infections in the general population, such as perineal hygiene and sexual practice, but these have not been investigated in patients receiving SGLT 2 inhibitors [52].

### 2.3. Pathophysiology

The pathophysiology of urogenital infections associated with SGLT 2 inhibitors is closely related to their mechanism of action, with these infections being considered the result of pharmacologically induced glycosuria, which provides a favorable environment for the growth of microorganisms.

Even if in vitro studies showed no difference in bacterial growth in the urine samples from diabetic patients with glycosuria and controls, the addition of glucose to urine samples significantly increased bacterial growth. In addition, in vitro studies demonstrated that glycosuria promotes the virulence of uropathogenic *E. coli*, a pathogen frequently isolated as an etiological agent in urinary tract infections [42].

Concerning genital mycotic infections, the most common pathogen is represented by *C. albicans*, a microorganism that possesses unique mechanisms including a glucose-inducible protein that promotes adhesion and impairs phagocytosis by the host, making it able to grow in a glucose-rich environment. In addition to this, it is well known that glucose is one of the growth-limiting factors of many Candida species [42,53].

The two mentioned infections, mycotic genital infections and urinary tract infections, differ slightly concerning their pathophysiology. Genital infections might be caused by an increased glucose quantity in the urine, which could be a substrate for microorganisms to grow on the genital epithelium. Also, in cases of vaginal infections, there is an impairment of the function of the vaginal epithelium to block the growth of microorganisms as a result of long-term diabetes, aging, hormonal imbalance, microvascular complications and decreased immunity [46]. Related to upper urinary tract infections, these might be caused by the reflux of bacteria from the lower urinary tract in the absence of renal abscess, renal papillary necrosis or renal emphysematous pyelonephritis, as long as asymptomatic bacteriuria in the lower urinary tract was associated with diabetes and it could be aggravated by increased glycosuria caused by SGLT2 inhibitors [46].

### 2.4. Clinical Implications

Clinical implications of SGLT2-associated urogenital infections are focused on three main aspects: the initiation of this class of drugs in the presence of risk factors for urogenital infections, the necessity of discontinuation of the treatment if such an infection develops while taking SGLT2 inhibitors, and the reinitiation of drugs after a urogenital infection.

Related to the first aspect, it is widely accepted that the cardio-reno-metabolic benefits of SGLT2 inhibitors generally exceed the risk of urogenital infections, even in patients with risk factors. Accordingly, SGLT2 inhibitor initiation is not contraindicated in patients with a history of uncomplicated urogenital infections or asymptomatic bacteriuria. Patients with recurrent or complicated infections should be evaluated in terms of underlying causes, and SGLT2 inhibitors could be safely initiated if these causes are resolved. Otherwise, in the presence of risk factors, preventive measures such as counseling on signs and symptoms of urogenital infections, as well as the maintenance of optimal local hygiene, should be emphasized. Not least, in diabetic patients, adequate glycemic control would decrease the risk of urogenital infections [54,55].

Concerning the development of urogenital infection during the treatment with SGLT2 inhibitors, in contrast to euglycemic ketoacidosis for which temporary discontinuation is advised, routine discontinuation of SGLT2 inhibitors in the setting of urogenital infections is not recommended; SGLT2 inhibitors should be continued in mild–moderate and clinically stable severe infections, while their discontinuation might be necessary for the setting of life-threatening infections when the risks significantly exceed the benefits [56]. This recommendation is sustained by large RCTs, in which patients who developed urinary tract infections did not stop the administration of the drug, and there was no increased risk of severe or recurrent infections compared to the placebo. At the same time, the important cardiovascular benefits of SGLT2 inhibitors dissipate after the interruption of the treatment [57].

Finally, in regards to the reinitiation of SGLT2 inhibitors after urogenital infection, this class of drugs should be reinitiated as soon as possible after the infection is treated and there are no other contraindications, considering that delaying SGLT2 inhibitors reinitiation might lead to clinical deterioration in previously stable patients by deprivation of a disease-modifying drug [42]. In patients at higher risk for urogenital infections, it is of great importance to monitor the signs and symptoms of urinary tract infections and genital mycotic infections, since the recurrence incidence is highest in the first two weeks after the reinitiation [58].

### 2.5. Clinical Manifestations and Management

The clinical manifestations of urogenital infections associated with SGLT2 inhibitors are extensive and various and their management depends on the type and severity of the infection, as well as on the context of the patients including comorbidities and other drugs taken simultaneously. Genital infections usually include balanoposthitis in men and vulvovaginal infections in women, while the spectrum of urinary tract infections ranges from asymptomatic bacteriuria, cystitis, pyelonephritis or severe urosepsis [30,59]. Additionally, diabetic patients are at an increased risk of complicated and severe urogenital infections such as emphysematous pyelonephritis, emphysematous cystitis, intrarenal abscess, candida infections and a more severe evolution of infections with Gram-negative pathogens apart from *E. coli* [60,61].

Considering the frequency and severity of urogenital infections in diabetic patients, prompt diagnosis and treatment are of great importance. The studies that followed the differences between antibiotic resistance rates of the strains isolated from diabetic patients compared with non-diabetic patients led to controversial results; thus, the choice of the antimicrobial agents should be based on the results of the laboratory susceptibility test, the context of the patient and the local resistance patterns, if the antibiotherapy is urgently needed [62].

Related to asymptomatic bacteriuria, there are few studies that recommend the treatment of this condition in order to prevent the risk of symptomatic urinary tract infections; current evidence does not sustain the antimicrobial treatment of asymptomatic bacteriuria since antibiotherapy does not seem to reduce complications in terms of time until the first symptomatic urinary tract infection, frequency of infections per 1000 days, hospitalization rates for such an infection or mortality [61]. Concerning other urinary tract infections, there is a lack of evidence regarding the most effective treatment regimen for acute cystitis and pyelonephritis in diabetic patients; the results from some studies concluded that an extended duration of treatment for cystitis was not associated with reduced recurrence rates, raising the question of whether several episodes of cystitis should be treated similar to those in non-diabetic people. Other studies led to different recommendations, supporting that these infections should be considered complicated urinary tract infections and treated for 7–14 days [62].

Regarding mycotic genital infections, in general, they are mild to moderate and respond well to topical or oral standard antifungal therapy [61,63].

## 3. Conclusions

SGLT2 inhibitors are a relatively new but valuable class of drugs that, besides the anti-hyperglycemic action, demonstrated cardiovascular and renal benefits, even in individuals without diabetes; consequently, treatment regimens combining SGLT2 inhibitors with other oral antidiabetic drugs represent an appropriate therapeutic option for patients presenting cardiovascular or renal comorbidities, and is recommended by current guidelines for those with a history of cardiovascular disease, or at high risk for it, as well as for patients with chronic kidney disease. With the increased use of this class of drugs, a heightened concern related to the risk of urogenital infections was raised. Even though SGLT2 inhibitors are in general well tolerated, their mechanism of action could lead to particular side effects; while these drugs increase the availability of glucose in the urinary tract, providing substrate for microorganisms to grow, they have been linked to urogenital infections. Thus, the Food and Drug Administration raised an alarm for severe urinary tract infections associated with SGLT2 inhibitors based on a limited number of reported cases; nevertheless, data from large randomized controlled trials showed no significant difference between SGLT2 inhibitors and the placebo. Considering the fact that most of these reports were based on symptoms rather than the results of the laboratory tests to prove infection, and the different reporting requirements for each trial, it is difficult to draw firm conclusions. Additionally, current evidence suggests that the risk of urogenital infections might be increased by the disease itself more than by the drugs.

While SGLT2 inhibitors have a well-established association with the risk of genital infections, the association with the development of urinary tract infections remains controversial and uncertain. Overall, in most cases, the benefits of this class of drug seem to outweigh the risk of infections, and this concern should not become a barrier in initiating therapeutic agents with so much potential for improving the condition of these patients.

## Data Availability

Not applicable.
